# News and Coming Events

**Published:** 1907-07-06

**Authors:** 


### NEWS AND COMING EVENTS.

THE next Norman Kerr Memorial Lecture, held in connection with the Society for the Study of Inebriety, will be delivered on the evening of Tuesday, October 8, 1907, by Dr. R. Welsh Branthwaite, H.M. Inspector under the Inebriates Acts.

THE King has conferred the title of "Royal" on the Bradford Eye and Ear Hospital. This institution was founded in 1857 by the late Dr. Edward Bronner, and will this year celebrate its Jubilee. It is now one of the largest special hospitals in the kingdom, and contains 46 beds. During last year 7,270 new patients were treated, 1,091 in-patients were admitted, and 1,084 major operations, including 160 extractions of senile cataract were performed. Since the foundation of the hospital no fewer than 158,848 cases have been treated, and 20,791 major operations performed. The present hospital is too small for the large number of in- and out-patients, and the committee is considering the question of building a large separate out-patient department.

THE Clinical Research Association, Ltd., which has lately moved into newly constructed premises at Watergate House, Adelphi, has signalised the event by the publication of a quarterly, "The Journal of Clinical Research." The new laboratories and offices are in an exceptionally fine situation at the top of a lofty building, whence they overlook South London to the Surrey Hills; they have been designed and fitted up throughout especially for the requirements of clinical pathology, and every detail betrays the most careful adaptation of means to ends. Though the bulkier furnishings are of British make, it is painful to learn that much of the glass work is from France; this is due to the absolute refusal of British manufacturers to carry out special designs invented by the staff to suit the exigencies of their work. System, order, and cleanliness are the dominant features of the work-rooms, and devices abound for reducing to vanishing point the possibility of infection of any of the laboratory staff.

AT an inquiry held recently at the Town Hall, West Bromwich, into an application by the Corporation to borrow £800 to extend the borough infectious diseases hospital, it was explained that the present administrative block at the hospital is insufficient for its uses. The accommodation for patients is sixty beds and twelve cots, and under the existing arrangements two nurses have to sleep in a downstairs sitting-room, two in the porter's lodge, and three maids in the isolation ward. Two schemes have been prepared, the first costing £800 and the second £1,000. The Sanitary Committee has adopted the cheaper scheme, and the proposed addition is suggested on the south side of the existing administrative block. The additional accommodation includes on the ground floor three bedrooms, one store-room, and one recreation-room, and on the first floor five bedrooms and one bathroom. It was explained that at the present time the hospital is serving West Bromwich, with a population of over 60,000, and Handsworth, with a similar population, and that the Handsworth authorities have an agreement to send small-pox cases to the hospital for two guineas per week, and scarlet fever cases for 30s. per week. The Government Inspector suggested that the scheme should include a discharging ward, as he considers the present arrangements are anything but satisfactory. It was mentioned that the approximate cost of adding an undressing-room, a small bathroom, and a small dressing-room would not be much more than £100, and the Inspector thought the Council ought to consider the advisability of doing something to improve the discharging arrangements of the hospital.

THE new wing of the East Ham Isolation Hospital, built at a cost of £6,250, has just been opened.

A TOTAL sum of £35,500 has been received to date in subscriptions for the Metropolitan Hospital Sunday Fund.

IN response to the appeal for £25,000 in aid of the funds of the Wigan Infirmary a sum of £4,566 16s. 6d. has been subscribed.

IN the July number of the *Caledonian Medical Journal* Dr. K. N. MacDonald contributes an interesting paper of recollections of "The Medical Giants of Edinburgh in the Early 'Fifties."

THE Medical Committee of the Cheltenham Spa is responsible for an attractive booklet on the merits of the Cheltenham mineral waters. A short introductory chapter gives some interesting historical notes on the Spa. The booklet contains valuable notes on the indications for using the baths and has an analytical table of the waters appended.

THE gas-heated hot-water circulators put on the market last summer by the Gas Light and Coke Company, Horseferry Road, S.W., have rapidly won popular approval, and the demand for them is steadily increasing. They can be fixed quite easily to existing flow and return pipes attached to an ordinary kitchen boiler, and the circulator and boiler can be used either separately or in conjunction.

MESSRS. WILSON AND STOCKALL, Bury, Lancashire, have received official information to say that they have been awarded the gold medal at the New Zealand International Exhibition, for their exhibit of a brougham ambulance. They have also received an order from the Municipality of Rome for one of their first-class accident ambulances, similar to that which they have recently supplied to the city of Milan.

THE difficulties of managing superstitious people are many. As the Hindu population object to killing rats, a Mr. Ram Narazan, a native banker, proposes to provide a "ratruksha," or sort of pen, in which captured rats may be confined as pensioners for the term of their natural lives, the sexes to be kept apart. The suggestion has been received gratefully by Major Buchanan, I.M.S., who is in charge of plague operations in India.

THE new City of London Lying-in Hospital, City Road, was opened on Monday last by H.R.H. Princess Christian. The hospital was instituted in 1750 and was removed to City Road in 1770. The reconstruction and refurnishing scheme has involved an expenditure of about £40,000, towards which only £3,000 has so far been received. This sum was paid by the Central London Railway Company as compensation for constructing their railway tubes so close to the hospital that in 1903 the London County Council condemned the institution buildings as unsafe.

THE next addition to the Oxford Medical Publications, to be ready next week, is "Operations in General Practice," by Messrs. Edred M. Corner and H. I. Pinches. The volume contains upwards of 175 illustrations, and it is meant to be a guide to the general practitioner in the performance of the more strictly surgical part of his daily routine. The first of the seven volumes of Professor Osler's "System of Medicine" is also almost ready for publication. It deals with predisposition and immunity, diseases caused by physical, chemical, and organic agents, by vegetable parasites, by protozoa, by animal parasites, nutrition, and constitutional diseases. It has been arranged that the volume shall be obtainable separately.

